# Clinical characteristics and survival of lung cancer patients associated with multiple primary malignancies

**DOI:** 10.1371/journal.pone.0185485

**Published:** 2017-09-28

**Authors:** Shan Shan, Jun She, Zhi-qiang Xue, Chun-xia Su, Shen-xiang Ren, Feng-ying Wu

**Affiliations:** 1 Department of Respirology, Shanghai sixth people's hospital, Shanghai Jiaotong University, Shanghai, China; 2 Department of Pulmonary Medicine, Zhongshan Hospital, Fudan University, Shanghai, China; 3 Department of Thoracic Surgery, PLA General Hospital, Shanghai, China; 4 Department of Oncology, Shanghai Pulmonary Hospital, Tongji University School of Medicine, Shanghai, China; Catalan Institute of Oncology, SPAIN

## Abstract

**Objectives:**

To investigate the characteristics and survival of lung cancer patients with additional malignant primary cancers.

**Methods:**

Records of lung cancer patients newly diagnosed in Shanghai Pulmonary Hospital between January 2000 and January 2010 were retrospectively reviewed. Patients with second primary lung cancer and those with lung cancer only were included for detailed analysis.

**Results:**

Of 27642 newly diagnosed lung cancer patients, 283 patients (1.02%) suffered previous additional primary cancers. Compared with single primary lung cancer, patients with secondary lung cancer associated other primary cancers were more often women (female to male ratio 1:1.72 vs 1:2.58, P = 0.018), older (64.2 vs 60.5 years old, P<0.001), more squamous cell type (30.7% vs 20.5%, P = 0.004), less small cell (3.9% vs 15.5%, P<0.001) type, at earlier stages (17.7% vs 11.0% for stage I, P = 0.014), and more frequently with family history of cancers (7.8% vs 3.9%, P = 0.038). The most common previous primary cancers observed were colorectal (22.0%), breast (18.4%), gastric (14.4%) and larynx cancers (11.9%). Approximately 42.9% of patients were diagnosed with lung cancer 2 to 6 years after diagnosis of initial primary cancers. The survival of patients with secondary lung cancer associated other malignancies was not significantly different from those with single lung cancer (P = 0.491), while synchronous multiple primary malignancies showed worse prognosis compared with those with metachronous ones or single lung cancer (p = 0.012).

**Conclusion:**

The possibility of second primary lung cancer should always be considered during the follow-up of related cancer types, especially those with family history of cancers. Patients with secondary lung cancer associated other primary malignancies have non-inferior survival than those with single lung cancer.

## Introduction

Multiple primary malignancies (MPMs) are defined as the formation of more than one different tumors in the same or different organs synchronously or metachronously. Early detection and improved treatments contribute to significantly prolonged survival time of cancer patients and allow more cancer patients to live long enough to develop subsequent primary cancers[1–3]. The occurrence of MPMs is related to genetic predisposition, exposure to same carcinogens, chemotherapeutic agents and radiotherapy for previous primary cancers[4]. Investigation of MPMs may help us further understand risk factors for second primary cancers.

Lung cancer is the leading cause of cancer mortality in the world[5–7]. Prolonged survival of cancer patients makes it possible to develop second primary lung cancer. Even though there is adequate published data describing the clinical characteristics of patients with MPMs, reports about lung cancer as a second primary cancer are rare. In the present study, we identified possible risk factors of development to lung cancer as a secondary primary cancer in MPMs and evaluated clinical characteristics and survival of patients with secondary lung cancer associated other MPMs.

## Materials and methods

### Patients

27642 lung cancer patients were newly diagnosed in Shanghai Pulmonary Hospital between January 2000 and January 2010. All cancer diagnosis was confirmed by histopathological examination. Of these patients, 283 cases were diagnosed other primary malignancies previously. 283 patients with single lung cancer within the same period were randomly selected as control group. We collected medical records and tumor registry information of these patients after institutional review board approval (Medical Ethics Committee of Shanghai Pulmonary Hospital). Epidemiological features, intervals and treatments for previous malignancies, family histories and prognosis were extracted and analyzed.

Second primary cancers diagnosed within 6 months after diagnosis of previous primary cancers were termed as synchronous primary cancers, and diagnosed beyond6 months were metachronous ones. Staging of lung cancer was determined by the seventh editions of the TNM classification system of the Union for International Cancer Control. Patients were classified into three groups according to the smoking status: never-smokers (0 pack year), light-smokers (<10 pack years), and smokers(≥10 pack years, including former smokers and current smokers). Family history of cancers was defined as cancer diagnosis in first-degree relatives. Occupational history, including exposure to asbestos or other known carcinogens, occupational exposure to dust or microscopic particles were also reviewed.

### Statistical analysis

All data was shown as means or as percentage within groups. Differences of clinical characteristics were analyzed by two-sided Fisher’s exact test. Categorical variables were analyzed by chi-square tests. Parameters included age, gender, smoking status, pathology, stage, and cancer history. The potential risk factors were analyzed with logistic regression models. Overall survival (OS) was calculated from the date of diagnosis to the date of death by Kaplan-Meier method. It was considered statistically significant if p value was less than 0.05. Statistical analyses were performed by SAS 9.3 (SAS Institute Inc., NC, USA) and SPSS 19.0 (IBM, NY, USA).

## Results

### Patient characteristics

Among the27642 newly diagnosed lung cancer patients between January 2000 and January 2010, 283 (1.02%) patients had one or more additional previously diagnosed malignancies. As shown in Table 1, clinical characteristics including mean age, gender, pathology, stage, smoking status and family history were compared between lung cancer associated with other MPMs group and single lung cancer group. The mean age of patients was 64.2 years old in MPMs group and 60.5 years old in single lung cancer group, showing significant difference (P<0.001). There were more female patients in MPMs group, with female to male ratio of 1:1.72 in MPMs group and 1:2.58 in single lung cancer group separately(P = 0.018). Regarding pathological types, an obviously higher incidence of squamous carcinoma (30.7% vs 20.5%, P = 0.004) and significantly lower incidence of small cell carcinoma (3.9% vs 15.5%, P<0.001) were observed in MPMs group. Furthermore, there were more stage I patients in MPMs group(17.7%)than in single groups (11%)(P = 0.014). Whereas no obvious difference was observed in stage II (7.4% vs 5.7%, P = 0.384), stage III (29.3% vs 31.8%, P = 0.518) or stage IV (45.6% vs 51.6%, P = 0.124). Additionally, patients with MPMs were prone to have family history of cancers (7.8% vs 3.9%, P = 0.038).Furthermore, no significant difference was found in smoking status (43.5% vs 41.0%, P = 0.540) between two groups.

**Table 1:** Clinical characteristics of patients with MPMs involving lung cancer and with single lung cancer.

	Multiple primaries (n = 283), n (%)	Single primary (n = 283), n (%)	P-value
Age at diagnosis			<0.001
Mean	64.2	60.5	
Range	28–86	29–82	
Gender			0.018
Male	179 (63.3%)	204 (72.2%)	
Female	104 (36.7%)	79 (27.8%)	
Pathology			
Adenocarcinoma	131 (46.3%)	129 (45.6%)	0.846
Squamous cell carcinoma	87 (30.7%)	58 (20.5%)	0.004
Small cell carcinoma	11 (3.9%)	44 (15.5%)	<0.001
Adeno-Squamous mixed carcinoma	21 (7.4%)	11(3.9%)	0.056
Large cell carcinoma	0 (0.0%)	2 (0.7%)	0.192
Non-small cell lung cancer	25 (8.8%)	34 (12.0%)	0.206
Others	8 (2.8%)	5(1.8%)	0.392
Stage			
I	50 (17.7%)	31 (11.0%)	0.014
II	21 (7.4%)	16 (5.7%)	0.384
III	83 (29.3%)	90 (31.8%)	0.518
IV	129 (45.6%)	146 (51.6%)	0.124
Smoking status			
Non—smoker	160 (56.5%)	167 (59.0%)	0.906*
light-smokers (<10 pack years)	43 (15.2%)	33 (11.7%)	
Smokers (≥10 pack years)	80 (28.3%)	83 (29.3%)	
Family history of cancer			0.038
No	261 (92.2%)	272(96.1%)	
Yes	22 (7.8%)	11(3.9%)	
Lung cancer	8(2.8%)	7(2.5%)	
Other cancers	14(4.9%)	4(1.4%)	
Occupational history			
Yes	21 (7.4%)	17 (6.0%)	0.561
No	262 (92.6%)	266 (94.0%)	

*non-smoker + light-smokers vs. smokers

### Additional primary malignancies in patients with lung cancer

**Fig 1:**
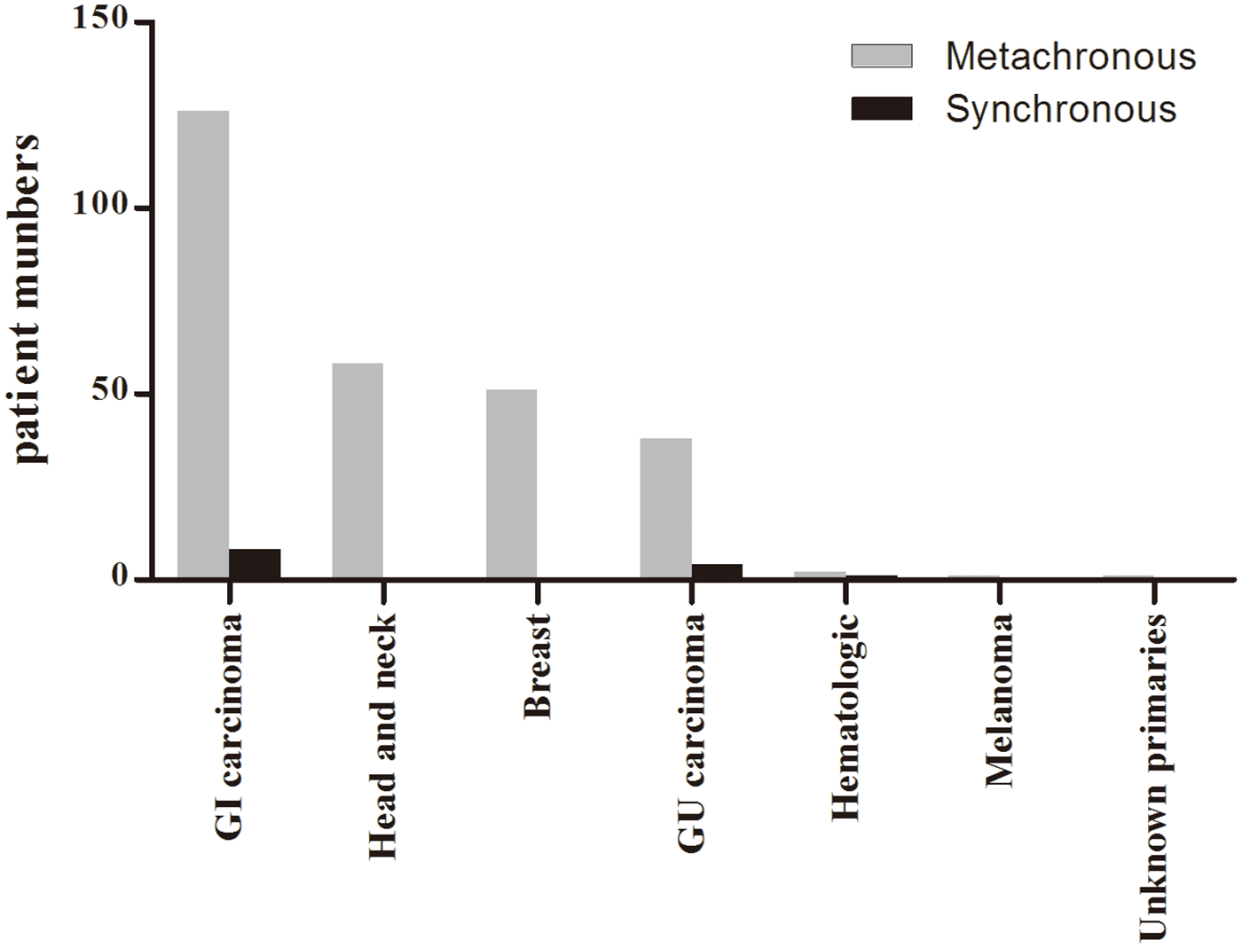
Types of additional malignancies in patients with MPMs associated lung cancer.

Of 283 MPMs patients, 15 patients (5.3%) had synchronous tumors and 268 (94.7%) had metachronous tumors. All patients had one previous primary cancer in the synchronous group. Whereas in the metachronous group, most of patients had one previous malignancy except 7 patients who had two previous primary cancers and 1 patient who had three previous malignancies. Number and types of previous primary cancers were shown in Table 2 and Fig 1. It was not surprising to find that colorectal (22.0%), breast (18.4%) and gastric (14.4%) cancer were the most common primary cancers in the metachronous MPMs group, since these cancers were of high morbidity in China. Interestingly, incidence of head and neck cancer accounted for 20.9%,of which laryngeal cancer (11.9%), the morbidity of which was1.2%-1.6% in China, presented to be fourth common cancer in lung cancer associated metachronous MPMs. Melanoma, cervical and pancreatic cancers represented to be the least frequent cancer types. Incidentally, none patients with prostate cancer and leukemia were observed in metachronous MPMs. In the synchronous group, colorectal, bladder and liver cancers were common cancers diagnosed synchronously with lung cancer. As for the small number of cases, we couldn’t conclude which cancers were more likely to accompany with lung cancer.

**Table 2:** Number and type of other primaries in MPMs associated with lung cancer.

Cancer types		Metachronous (n = 268),n (%)	Synchronous n (%) (n = 15), n (%)
GU carcinoma	Total	38 (13.7%)	4 (26.7%)
	Kidney	11 (4.0%)	
	Pelvis	3 (1.1%)	
	Bladder	11 (4.0%)	3 (20.0%)
	Cervix	8 (2.9%)	
	Ovary	1 (0.4%)	
	Uterus	4 (1.4%)	1 (6.7%)
GI carcinoma	Total	126 (45.5%)	8 (53.3%)
	Colorectal	61 (22.0%)	5 (33.3%)
	Gastric	40 (14.4%)	1 (6.7%)
	Esophagus	10 (3.6%)	
	Pancreas	2 (0.7%)	
	Liver	9 (3.2%)	2 (13.3%)
	Cholecyst and Biliary ducts	2 (0.7%)	
	Other intestinal tract	2 (0.7%)	
Breast		51 (18.4%)	
Head and neck	Total	58 (20.9%)	1 (6.7%)
	Thyroid	12 (4.3%)	1 (6.7%)
	Larynx	33 (11.9%)	
	Nasopharyngeal	10 (3.6%)	
	Oral	3 (1.1%)	
Hematologic	Total	2 (0.7%)	1 (6.7%)
	Lymphoma	2 (0.7%)	1 (6.7%)
Melanoma		1 (0.4%)	
Skin			1 (6.7%)
Unknown primaries		1 (0.4%)	

GI, gastrointestinal; GU, genitourinary.

Three primaries: 7 cases

Four primaries: 1 case

### Intervals between lung cancer and additional primaries

**Fig 2:**
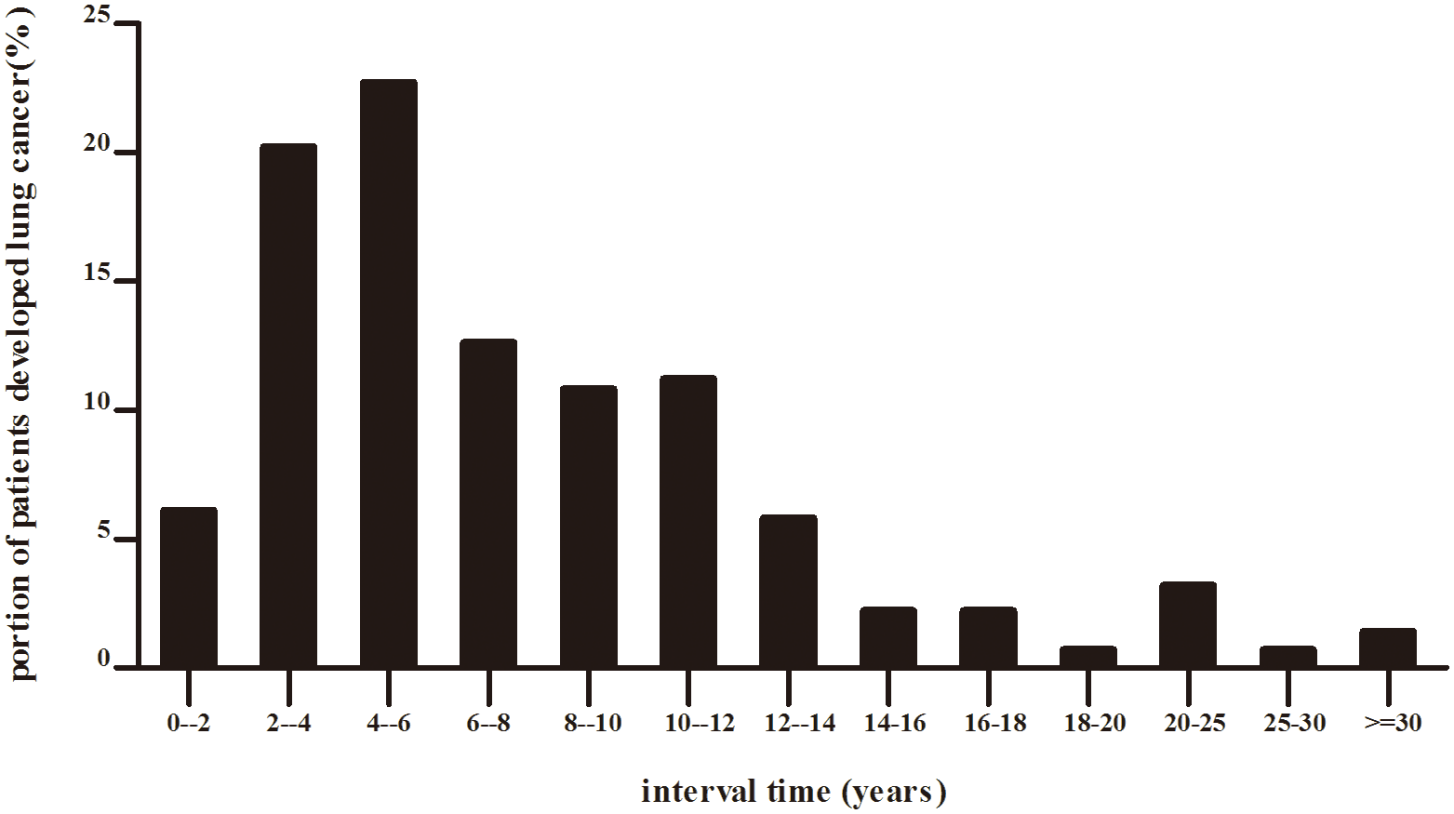
Proportion of patients with first primary cancer developed lung cancer within different intervals.

In our study, the mean and median interval time between previous primary cancer and lung cancer was 7.4±5.9 years and 6 years respectively, ranging from 0.75 to 40 years in metachronous group. As shown in Table 3 and Fig 2, 42.9% of MPMs patients developed lung cancer 2 to 6 years after diagnosis of first primaries. Chances of developing lung cancer decreased as time went by. The average intervals of different cancer types developing secondary lung cancer were investigated. Result showed that gastric, thyroid and breast cancer presented to have longest interval time, with the intervals of 9.3±3.0, 9.0±3.0 and 8.1±2.8 years separately, which was obviously longer than cervical and liver cancer(3.9±1.9 and 3.2±1.8 years, P<0.05).

**Table 3:** Average interval time for different cancer type to develop MPMs associated lung cancer.

Metachronous interval time(years)	Number	Percentage
0–2	17	6.10%
2–4	56	20.20%
4–6	63	22.70%
6–8	35	12.60%
8–10	30	10.80%
10–12	31	11.20%
12–14	16	5.80%
14–16	6	2.20%
16–18	6	2.20%
18–20	2	0.70%
20–25	9	3.20%
25–30	2	0.70%
> = 30	4	1.40%
Total	277	

### Treatments of previous malignant primaries

We analyzed treatments for previous primary cancers among metachronous MPMs patients. In these 267 cases, 254 (95%), 91 (34%), 45 (17%) and 10 (4%) patients received surgery, chemotherapy, radiotherapy and endocrine therapy respectively. Those who underwent chemotherapy were more likely to be gastrointestinal cancer patients (47/91, 51.6%), and those who underwent radiotherapy were more likely to be head and neck cancer patients (20/45, 44.4%) and breast cancer patients (10/45, 22.2%). When we separated treatments into chemotherapy alone, radiotherapy alone and chemo-radiotherapy, it was interesting that more squamous cancer and less adenocarcinoma cell in radiotherapy alone group than in chemotherapy alone group (37% vs 32% for squamous carcinoma, 42% vs 51% for adenocarcinoma separately, P>0.05), but it failed to reach statistical significance.

### Survival of patients with MPMs involving lung cancer

**Fig 3:**
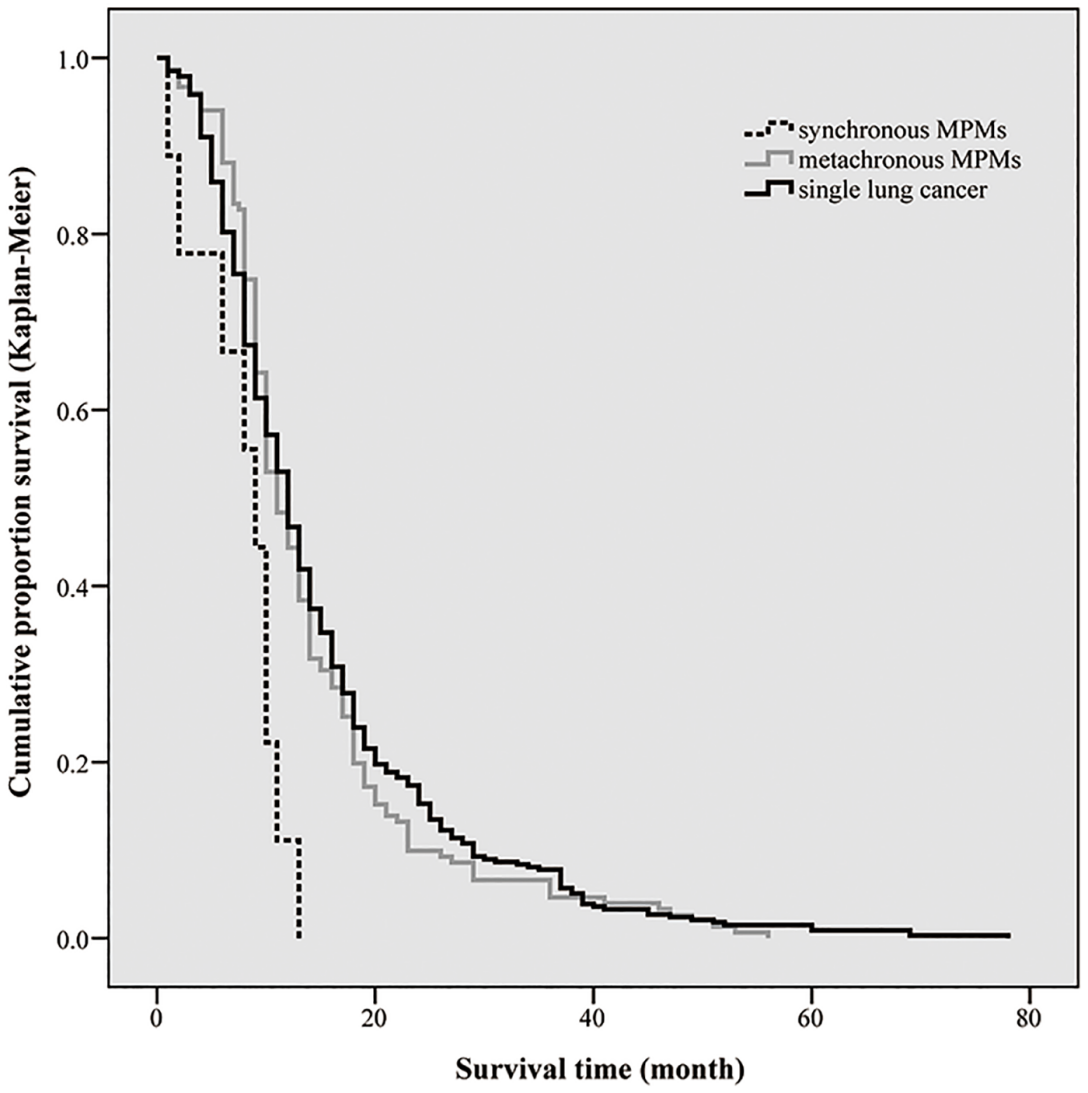
Survival of patients between metachronous group and synchronous group. Difference between two groups was statistically significant (P = 0.001).

We further investigated the prognosis of lung cancer patients with previous primary cancers. Survival data of 151 patients in metachronous group (calculated from diagnosis of lung cancer), 9 patients in synchronous group and 254 patients in single lung cancer group were analyzed separately. As shown in Fig 3, no significant difference was found in the overall survival for patients in metachronous group when compared with single lung cancer group, with the median survival of 14.32 months and 15.14months separately (P = 0.491). However, patients with synchronous primary cancers had obvious inferior survival when compared with those with metachronous primary cancers or single lung cancer (median OS: 7.78, 14.32 and 15.14 months, P = 0.012).

## Discussion

Cancer survivors have a 14% increased risk of developing a second primary cancer compared with the general population[8]. The incidence of MPMs has increased due to the prolonged survival of cancer patients, increased awareness of the possibility of second malignancies, and frequent use of sensitive screening procedures[4]. It was reported that the overall prevalence of MPMs was between 0.73% and 11.7%[9–11]. But there were no reports addressed the incidence of lung cancer as a second primary malignancy. In the present study, we retrospectively analyzed the secondary lung cancer associated other MPMs in ten years and found that the incidence of MPMs was 1.02%among all the lung cancer patients in Chinese patient population.

### Characteristics of lung cancer involved MPMs

Previously, Watanabe et al reported that squamous cell carcinoma was the most frequent histologic type of lung cancer in patients with multiple primary malignancies between 1962 and 1981[12]. Liu et al. found that adenocarcinoma was the most frequent histologic type of lung cancer with MPMs[13]. In our study, we found that adenocarcinoma presented to be the most frequent histologic type, which was similar to Liu’s study.

In terms of stage, stage I showed higher percentage in MPMs group, which may be attributed to the routine follow-up of previous malignancy and increased awareness of symptoms.

### Possible risk factors for a second primary lung cancer associated with other MPMs

Carcinogenesis is a multi-step and complex process. The development of MPMs might be attributed to shared exposure to risk factors including environment, lifestyle, inherited genes, or treatments of initial primary cancers. To investigate the possible risk factors for later primary lung cancer, we analyzed age, gender, smoking status, genetic factors, previous cancer types, occupational history, intervals and treatments for previous primary cancers in MPMs patients.

Age is considered to be a risk factor for MPMs. The prevalence of MPMs was reported to be 5–12% for patients aged 50–64 years old, compared with 12–26% for those aged over 80 years old[1]. Geryk et al found that the highest burden of MPMs was among patients aged 50–69 years old[14]. In the present study, the average age of secondary lung cancer associated MPMs was 64.2 years old, which was significantly higher than single lung cancer group (60.5 years old), indicating that age added the risk of development of secondary lung cancer.

Tobacco smoking has been proved to increase the risk of MPMs. Cancer survivors with smoking history had a higher risk of MPMs compared with non-smokers[1, 15]. However, in our analysis, we didn’t find any differences in smoking status between secondary lung cancer associated MPMs and single lung cancer patients.

It is well known that patients with family history of cancers would inherit genetic cancer susceptibility, which increases the risk of MPMs[16–18]. For example, microsatellite instability was noticed to more frequently occur in cases of MPMs than sporadic cancers[19–20].In the present study, MPMs patients with secondary lung cancer were more often with history of cancers. It was reported that impaired DNA damage repair contributed to the formation of non-small cell lung cancer in MPMs patients since benzopyrene diol epoxide-induced DNA damage were significantly higher in patients with MPMs[18]. It still needed further investigations on specific genes involved in these processes.

Certain cancers might be associated with the increased risk of subsequent primary lung cancer. Our results showed that patients who initially presented with colorectal, breast, gastric and head and neck cancers were more liable to develop second primary lung cancer. Furthermore, malignancies such as oral cavity, pharynx, larynx and lung cancers were reported in patients previously diagnosed with similar malignancies because of smoking exposure[2, 21–22]. Interestingly, head and neck cancer, especially laryngeal cancer, had strong association with second primary lung cancer. Previously, Michael et al. found that patients with head and neck squamous cell carcinoma were at high risk of developing second primary lung cancer, with the risk of 5.8%, 11.4%, and 16.4% at 5, 10, and 15 years after diagnosis of head and neck cancer respectively. Smoking exposure and/or host factors may lead to obvious association between these two cancer types[23]. Our recent data further validated these strong associations and suggested that close monitoring should be carefully conducted in patients harboring these malignancies.

Time intervals might be associated with the onset of secondary lung cancer. Liu et al reported that about 59.9% of secondary lung cancer occurred within 5 years after the initial diagnosis of the first cancer[13]. Scott et al reported that the risk for lung cancer may be elevated within the first 4 years ofthe treatment of Hodgkin’s disease[24], while others reported that the increased risks is not apparent until the fifth year[25]. Our study showed that a big proportion of secondary lung cancers were diagnosed within the 2nd to 6th years after the diagnosis of first malignancy. Therefore, careful follow-up are necessary during this treatment interval.

Briefly, genetic predisposition, previous cancer treatments, smoking, lifestyles, and other aspects could act as risk factors in the developments of secondary lung cancer.

### Survival analysis

Survival of patients with multiple primary cancers varies depending on the method of calculation and primary tumor types. Some studies reported no differences in survival when comparing MPMs with single cancers[26–27], while some other reported worse prognosis for patients with multiple primary cancers when calculating survival from the date of the second primary cancers[11]. For lung cancer as a second primary cancer, the 5-year survival for those with metachronous primary cancers was 44% compared with 10% for those with synchronous primary cancers[13]. In the present analysis, survival of lung cancer associated with other MPMs patients showed no significant difference with that of single lung cancer patients. However, patients with synchronous cancers showed obviously worse survival than those with metachronous cancers or single ones. Heavy tumor burden in synchronous lung cancer patients may contribute to the worse prognosis.

In summary, secondary lung cancer associated other MPMs were more often women and older, and those with family history of cancers. Second primary lung cancer was more likely to develop in patients with initial primary cancers such as colorectal, breast, gastric and larynx cancer, 2 to 6 years after the first primary malignancy diagnosis. Therefore, careful follow-up are necessary during this treatment interval for patients with these cancers. Furthermore, previously primary cancers did not seem to adversely impact the survival of patients with second lung cancer, suggested that these patients should be treated appropriately and timely, especially those with synchronous lung cancer patients.

## Supporting information

S1 File Original data of our study. (XLS)
